# Implementation of an AI-supported decision-making tool in a high-volume stroke system with routine perfusion imaging

**DOI:** 10.1093/esj/aakag098

**Published:** 2026-08-20

**Authors:** Adrian Karlsson, Erik Cadeborn, Malin Woock, Arne Allardt, Katarina Jood, Isabella Björkman-Burtscher, Alexandros Rentzos

**Affiliations:** Department of Radiology, Institute of Clinical Sciences, Sahlgrenska Academy, University of Gothenburg, Gothenburg, Sweden; Section of Diagnostic and Interventional Neuroradiology, Department of Radiology, Sahlgrenska University Hospital, Region Västra Götaland, Gothenburg, Sweden; Section of Diagnostic and Interventional Neuroradiology, Department of Radiology, Sahlgrenska University Hospital, Region Västra Götaland, Gothenburg, Sweden; Department of Clinical Neuroscience, Institute of Neuroscience and Physiology, Sahlgrenska Academy, University of Gothenburg, Gothenburg, Sweden; Department of Neurology, Sahlgrenska University Hospital, Region Västra Götaland, Gothenburg, Sweden; Department of Clinical Neuroscience, Institute of Neuroscience and Physiology, Sahlgrenska Academy, University of Gothenburg, Gothenburg, Sweden; Department of Neurology, Sahlgrenska University Hospital, Region Västra Götaland, Gothenburg, Sweden; Department of Clinical Neuroscience, Institute of Neuroscience and Physiology, Sahlgrenska Academy, University of Gothenburg, Gothenburg, Sweden; Department of Neurology, Sahlgrenska University Hospital, Region Västra Götaland, Gothenburg, Sweden; Department of Radiology, Institute of Clinical Sciences, Sahlgrenska Academy, University of Gothenburg, Gothenburg, Sweden; Section of Diagnostic and Interventional Neuroradiology, Department of Radiology, Sahlgrenska University Hospital, Region Västra Götaland, Gothenburg, Sweden; Department of Radiology, Institute of Clinical Sciences, Sahlgrenska Academy, University of Gothenburg, Gothenburg, Sweden; Section of Diagnostic and Interventional Neuroradiology, Department of Radiology, Sahlgrenska University Hospital, Region Västra Götaland, Gothenburg, Sweden

**Keywords:** artificial intelligence, endovascular treatment, stroke

## Abstract

**Introduction:**

Endovascular thrombectomy (EVT) is the standard of care for LVO stroke; however, rapid workflow is critical for a beneficial outcome. Artificial intelligence (AI)-supported imaging tools, such as Brainomix 360 Stroke, may improve the efficiency of acute stroke imaging and reduce treatment delays. We evaluated the effects of regional implementation of Brainomix 360 Stroke in a high-volume system with routine use of perfusion imaging.

**Patients and methods:**

We conducted a retrospective register-based cohort study of consecutive patients treated with EVT at Sahlgrenska University Hospital from June 2021 to May 2024. Patients outside the Region Västra Götaland prehospital area or aged under 18 years were excluded. The study period was divided into pre-implementation, learning and established periods following Brainomix 360 Stroke introduction. Primary organisational outcomes were time from non-contrast brain CT (NCCT) to CT perfusion map availability (CT-to-perfusion-map-availability) and from NCCT to groin puncture (CT–puncture). Clinical outcomes included early neurological improvement (≥4 points on the NIHSS or a score of 0–1 at 24 h) and favourable functional outcome (mRS scores 0–2 or return to pre-stroke mRS at 90 days).

**Results:**

A total of 970 EVT patients were included. Adjusted median CT–puncture significantly decreased from 47 min pre-implementation to 36 and 35 min in the learning and established periods, respectively. Adjusted median CT-to-perfusion-map-availability significantly decreased from 7 min pre-implementation to 6 min in the established period. In subgroup analyses of primary stroke centres (PSCs), results were similar while no significant differences were found in subgroup analyses of the comprehensive stroke centre. No significant differences were observed in clinical outcomes.

**Discussion and conclusion:**

Implementation of an AI-supported imaging decision-making tool was associated with significant reductions in key workflow times, largely attributable to decreased times at PSCs in the region.

## Graphical Abstract

**Graphical Abstract:**
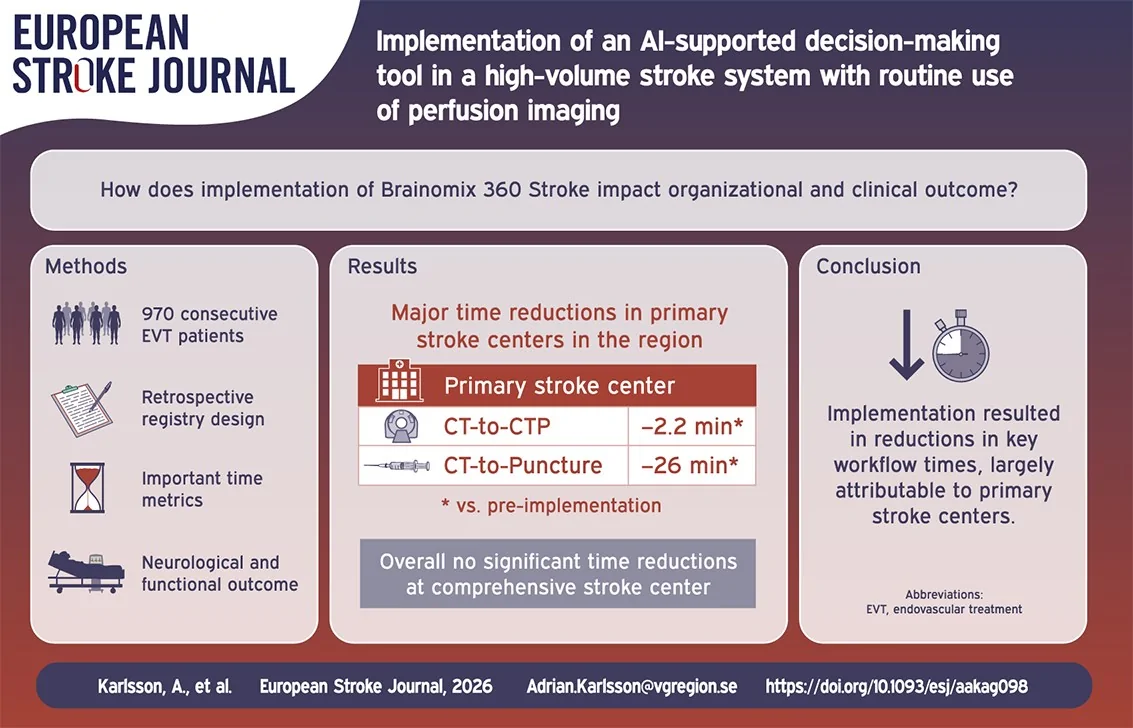

## Introduction

Endovascular thrombectomy (EVT) in acute ischaemic stroke (AIS) has been demonstrated to provide substantial benefits compared with best medical treatment in patients with LVO when performed within 24 h of symptom onset.^1^^,^^2^ Endovascular thrombectomy is now regarded as the standard of care in LVO stroke. However, time to treatment remains a critical determinant of patient outcome, and multiple components of the stroke care pathway have been evaluated and optimised to minimise delays. One such component is AIS imaging, where artificial intelligence (AI)-supported tools have been proposed to improve efficiency and reduce delays.^3^

Sahlgrenska University Hospital is a comprehensive stroke centre (CSC) serving the 6 acute primary stroke centres (PSCs) of Region Västra Götaland in Sweden with a total population of 1.8 million. In June 2022, Brainomix 360 Stroke was introduced across the region. Brainomix 360 Stroke is designed to automatically detect and quantify key imaging features in acute stroke. In our region, non-contrast CT (NCCT), CTA and CTP are routinely used, with Brainomix 360 Stroke providing automated detection of bleeding and infarcted parenchyma on NCCT, automated clot detection on CTA and quantitative perfusion maps on CTP.

Previous research has primarily focused on the diagnostic performance and accuracy of various AI-based decision-making tools.^4–6^ In contrast, evidence of their impact at the organisational level within stroke care systems remains limited. While a few studies have reported significant reduction in workflow time metrics, these studies have typically excluded patients presenting to PSCs and/or included small numbers of EVT-treated patients and have made limited use of CTP.^7^^,^^8^ Our study aims to evaluate the effect of implementing an AI-supported imaging decision-making tool within an experienced high-volume stroke care system with routine use of advanced perfusion imaging prior to treatment.

## Patients and methods

### Study design and data sources

This retrospective, register-based cohort study included all consecutive patients with MeVO or LVO stroke who underwent EVT at Sahlgrenska University Hospital (SUH), the only CSC of Västra Götaland Region (VGR) from June 2021 to May 2024. Patients younger than 18 years, from outside the VGR prehospital area, and those from centres not considered stroke centres (ie, only managing in-hospital strokes in patients admitted for other reasons and lacking an established routine stroke workflow) were excluded. Patients were identified through the local research registry, the Sahlgrenska Stroke Recanalization Registry (SSRR), which collects data for all consecutive patients treated with EVT at SUH. The SSRR integrates clinical and procedural data from the national EVT registry (EVAS, https://evas-registret.se/, coverage rate 97%) and the Swedish Stroke Register (Riksstroke, https://www.riksstroke.org/eng/, coverage rate 95%) based on the unique Swedish personal identification number (PIN). Other relevant clinical data, not available in the aforementioned national registries, are collected from local electronic medical records and linked to the SSRR using the same unique Swedish PIN.

Prehospital transport-related data were retrieved from the national prehospital ambulance electronic records Ambulink and SOS-alarm. Data from Brainomix 360 Stroke were extracted from both local and company servers. All data were linked to the SSRR data by using the unique Swedish PIN or, in the case of server data, a pseudonymised case ID. A detailed description of all included variables, including origin and validity, is presented in Table S1.

### Endovascular treatment and group definitions

Patients were eligible for EVT if they presented with clinical symptoms of LVO or MeVO in the anterior or posterior circulation ischaemic stroke, confirmed by CTA upon hospital arrival, and if treatment could be initiated within 24 h of symptom onset. Patients without contraindications who arrived at a PSC or CSC within 4.5 h from symptom onset also received intravenous thrombolysis (IVT). Intravenous thrombolysis in the extended window (>4.5 h after stroke onset) was not part of routine clinical practice in our region during the study period. Advanced multimodal imaging (NCCT, CTA and CTP) was routinely performed. All EVT procedures were perioperatively managed by a dedicated neuroanaesthesiology team. Off-hours were defined as 16:15–07:30 on weekdays and all day during national holidays and weekends. During off-hours neurointerventional staffing was limited to one complete team (consultant, nurse and assistant nurse), on call from home, with an activation time of less than 30 min. In contrast, during working hours, the neurointerventional team was present on site. Otherwise, the stroke workflow regarding prehospital, radiology, anaesthesiology and neurology procedures does not differ between working and off-hours, but staff on call during off-hours at PSCs may be less experienced compared with those on call during working hours. Late window treatment was defined as 6–24 h from stroke insult to groin puncture. Prior to the study period, a number of measures had already been implemented to optimise the workflow for acute stroke, including allowing the primary prehospital transport unit to remain on standby during acute imaging at the PSC to then directly continue as secondary transport to the CSC, potentially reducing time delays relating to acquiring a secondary transport. A detailed description of workflow modifications implemented before the study period is given in Table S2. No other major changes to the stroke workflow apart from the AI tool were implemented during the studied period.

### Implementation of AI-supported acute stroke imaging diagnostics

In June 2022, Brainomix 360 Stroke was introduced simultaneously across all hospitals in the Region Västra Götaland including the CSC (Sahlgrenska University Hospital). Implementation involved on-site training and e-learning resources.

The study period was divided into 3 periods:

- **Control period (period 1)**: the year preceding Brainomix 360 Stroke implementation.
- **Learning period (period 2)**: the first year after implementation.
- **Established period (period 3)**: the second year after implementation.

### Outcome measures

**Organisational outcomes** were:

- Time from NCCT to when CTP maps were available to the neurologist (**CT-to-perfusion-map-availability**), reflecting internal radiology workflow.
- Time from NCCT to groin puncture (**CT-to-puncture**), consisting of the interval from initial imaging at a PSC or CSC to EVT initiation at the CSC reflecting post-imaging decision-making and transport delay.

Imaging time was extracted manually from DICOM data in the local PACS (pre-implementation) and from Brainomix local and remote servers (post-implementation). The CT-to-perfusion-map-availability time was defined as the time from NCCT scan acquisition to the availability of the perfusion maps in the PACS pre-implementation and to the availability of the AI-generated perfusion report in the AI tool application post-implementation. Time of groin puncture was obtained from the SSRR. In our regional stroke workflow, patients are transported by ambulance directly to the CT scanner, bypassing the emergency department, so that NCCT time is equivalent to door-in time. Moreover, the ambulance crew transporting the patient to the PSC waits 20 min after arrival at the CT scanner for possible secondary transfer to the CSC, while the neurology and radiology staff are already present at the scanner. According to regional guidelines, the neurologist examines the patient upon arrival and may interrupt the examination when the radiology team is ready to start imaging. The neurological examination continues in parallel if needed. If no contraindications are present IVT is administered either before or during CTA and CTP acquisition. The radiologist reports the findings of NCCT, CTA and CTP sequentially directly to the neurologist. After AI implementation, the AI tool report was used in addition to the radiologist’s assessment, either confirming the findings or identifying occlusions missed on CTA by the radiologist, while also providing automated perfusion maps without manual post-processing. Radiological images (scrollable image stacks), automated perfusion maps and the generated report are available immediately to the on-call neurologist through the Brainomix 360 Stroke app, regardless of where in the region the patient undergoes imaging. The software also includes a communication platform and the ability to flag patients as potential candidates for EVT; however, these features are not used in our region. Computed tomography-to-perfusion-map-availability was chosen as the primary outcome metric of internal workflow efficiency because it captures the full internal workflow, including image acquisition and parallel communication between radiologist and neurologist, and can also be applied across all centres in the region, unlike door-in-door-out times, which apply only to referring centres. Moreover, it offers high data completeness and quality.

**Clinical outcomes** included:

- Early neurological improvement (**ENI**), defined as a ≥ 4-point reduction in NIHSS or an absolute NIHSS of 0–1 at 24 h post-intervention.
- Favourable functional outcome (**FFO**), defined as an mRS score of 0–2, or return to pre-stroke mRS, at 90 days.

The definition of ENI was selected as we included both LVO and MeVO patients. The higher threshold of 8 points used in some studies may fail to detect clinically important improvement in patients with fewer symptoms at baseline. Previous studies have shown that this definition of ENI is associated with improvement in functional outcome and might provide an optimal threshold at 24 h.^9^

### Statistical analysis

All statistical analyses were performed using **RStudio (version 2025.05.0 + 496)**. The distribution of continuous variables was assessed using histograms and the Kolmogorov–Smirnov test for normality. Data are presented as mean (SD) for normally distributed continuous variables, median (IQR) for skewed variables and number (percentage, %) for categorical variables.

Baseline imbalances between groups were evaluated using standardised mean differences (SMDs).^10^ Variables with SMD ≥ 0.1 were considered potential confounders and included in multivariable models (stroke within 2 weeks prior to current event, IV-rtPA, general anaesthesia, occlusion location, infarct > 1/3 of media territory at baseline and receiving stroke centre).

For CT-to-puncture and CT-to-perfusion-map-availability, the Kruskal–Wallis test was applied with pairwise comparisons using Dunn’s test corrected for multiple testing using the Bonferroni method. Adjusted continuous outcomes were analysed using quantile regression at the median (tau = 0.5). For clinical outcomes, odds ratios (ORs) were estimated using logistic regression including potential confounders as above with the control period as the reference. To account for centre clustering, the referring hospital was included as a fixed-effect covariate. A random intercept model (lqmm) was considered but found to be numerically unstable given the small number of centres (7 in total) and highly unequal cluster sizes. In sub-analyses of CSC-only, no adjustment for centre (individual PSCs and CSC) were made.

Approximately 10% of observations had missing values in variables adjusted for. To avoid listwise deletion and potential selection bias, missing values were handled using multiple imputation by chained equations, implemented with the mice package in R. Twenty imputed datasets were generated with 10 iterations each. Quantile regression models were fitted separately on each imputed dataset, and results were pooled using Rubin’s rules, with standard errors derived from bootstrap resampling (200 resamples per imputed dataset). The imputation model included all variables used in the adjusted analyses, including study period and receiving stroke centre, to preserve temporal structure and centre-level differences. Outcome variables were included as predictors in the imputation process but were not themselves imputed. Confidence intervals and *P*-values were calculated using Barnard–Rubin–adjusted degrees of freedom.

Prespecified subgroup analyses were performed according to referring hospital type (CSC vs PSC) and presentation during working hours vs off-hours. These subgroup analyses were considered exploratory and were undertaken to assess the consistency of findings across clinically relevant settings. No formal adjustment for multiple testing was applied because of the increased risk of type I error; therefore, subgroup findings should be interpreted cautiously and regarded as hypothesis-generating.

An a posteriori calculation of the minimum detectable OR was performed for the main analysis and subgroups using the observed sample size, event rate and *a* = 0.05 (Table S3). Patients from referring hospitals not considered stroke centres were excluded (6 patients). To assess the robustness of the imputation procedure, a complete-case analysis comparison was performed using the same model specification as in the primary analyses (Table S4).

All analyses except the a posterior minimum detectable OR estimate analysis and multiple imputation vs complete case comparison were planned prior to the study start. A 2-sided *P*-value of ≤ .05 was considered significant.

## Results

**Figure 1:**
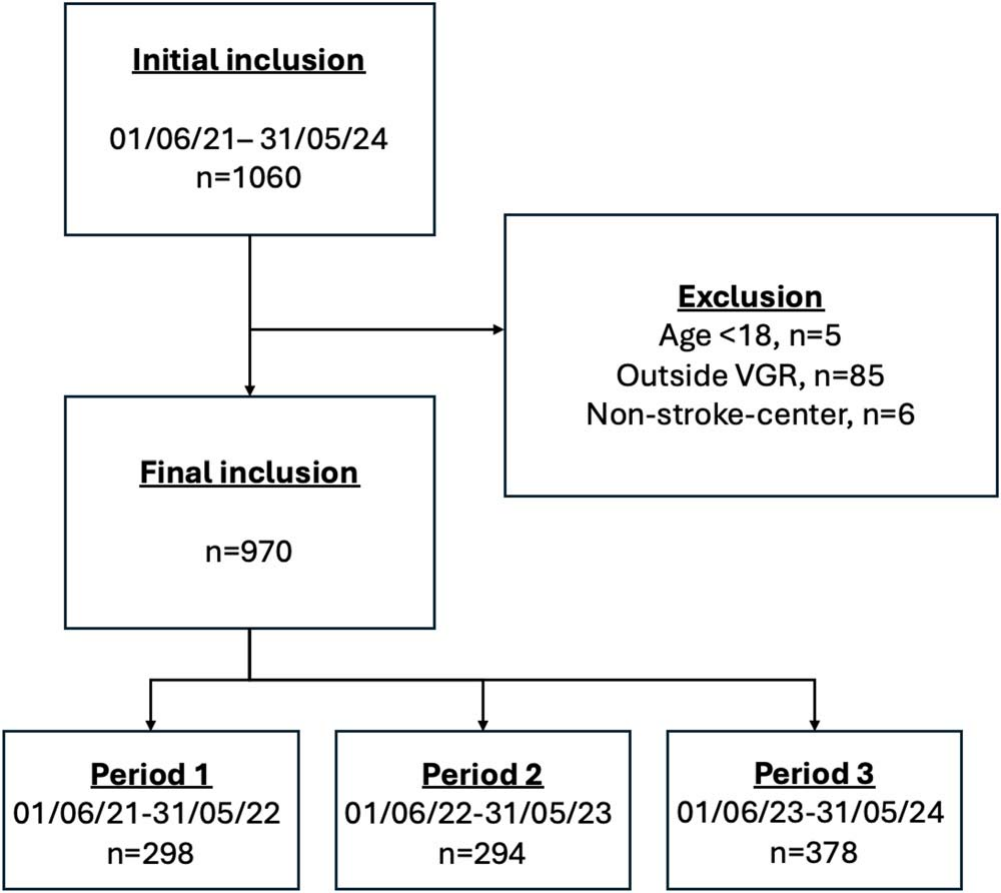
Flowchart of patient selection. Abbreviations: PSC = primary stroke centre; VGR = Västra Götalandsregionen.

Cohort selection is illustrated in the inclusion flow-chart in Figure 1. This study covers 970 patients treated with EVT during a 3-year period (June 2021–May 2024). Baseline data of included patients are shown in Table 1.

**Table 1:** Baseline characteristics, current event and procedural data per period.

Variable	*N*	Period 1 *N* = 298 ^a^	Period 2 *N* = 294 ^a^	Period 3 *N* = 378 ^a^	SMD
**Sex, male**	968	153 (51.3%)	149 (50.7%)	202 (53.7%)	0.05
**Age, y**	970	77.0 [68.0, 83.0]	78.0 [70.0, 85.0]	78.0 [70.0, 85.0]	0.08
**SBP before intervention, mmHg**	926	147.0 [130.0, 165.0]	146.0 [130.0, 160.0]	150.0 [134.0, 164.0]	0.09
**Fully conscious before intervention**	970	270 (90.6%)	268 (91.2%)	340 (89.9%)	0.02
**Transfer from another hospital**	970	130 (43.6%)	118 (40.1%)	171 (45.2%)	0.07
**IV thrombolysis before intervention**	970	105 (35.2%)	92 (31.3%)	102 (27.0%)	0.18
**Stroke within 2 weeks prior**	935	14 (4.8%)	30 (10.8%)	30 (8.2%)	0.23
**Pre-stroke mRS 0–1**	913	201 (72.0%)	195 (71.4%)	268 (74.2%)	0.05
**NIHSS before intervention**	931	15.0 [8.0, 20.0]	14.5 [9.0, 20.0]	15.0 [9.0, 19.0]	0.05
**General anaesthesia**	970	198 (66.4%)	221 (75.2%)	322 (85.2%)	0.42
**Late window (6–24 h)**	877	91 (27.8%)	83 (25.2%)	99 (25.8%)	0.08
**Referring hospital**	970				0.34
**CSC**		168 (56.4%)	178 (60.5%)	205 (54.2%)	
**PSC 1**		26 (8.7%)	22 (7.5%)	41 (10.8%)	
**PSC 2**		17 (5.7%)	9 (3.1%)	5 (1.3%)	
**PSC 3**		47 (15.8%)	38 (12.9%)	52 (13.8%)	
**PSC 4**		20 (6.7%)	21 (7.1%)	45 (11.9%)	
**PSC 5**		8 (2.7%)	12 (4.1%)	5 (1.3%)	
**PSC 6**		12 (4.0%)	14 (4.8%)	25 (6.6%)	
**Occlusion site**	911				0.23
**ICA**		63 (22.3%)	74 (26.6%)	104 (29.6%)	
**M1**		109 (38.7%)	100 (36.0%)	122 (34.8%)	
**M2**		72 (25.5%)	74 (26.6%)	86 (24.5%)	
**M3/Anterior**		10 (3.5%)	6 (2.2%)	18 (5.1%)	
**Posterior**		28 (9.9%)	24 (8.6%)	21 (6.0%)	

Abbreviations: CSC = comprehensive stroke centre; IV = intravenous; PSC = primary stroke centre; SBP = systolic blood pressure; SMD = standardised mean difference (maximum pairwise across periods).

Patients with initial imaging at non-stroke-centres were excluded.

^a^Data are presented as median [IQR] or *n* (%).

### Organisational outcome measure

Computed tomography-to-perfusion-map-availability data were available for 879 patients (90%). The Kruskal–Wallis test did not indicate a statistically significant difference across periods (*P* = .136) before adjustment. In adjusted analyses, the median CT-to-perfusion-map-availability time decreased significantly from 7 min in the pre-implementation period to 6 min in the established period (*P* = .025). In analyses restricted to PSC, the adjusted median CT-to-perfusion-map-availability time decreased significantly from 9 min in the pre-implementation period to 6 min in the established period (*P* = .008).

Computed tomography-to-puncture data were available for 921 patients (95%). The Kruskal–Wallis test showed a statistically significant difference across periods (*P* < .001) before adjustment. Pairwise comparisons demonstrated significant differences between period 1 and period 2 (*P* < .001) and between period 1 and period 3 (*P* = .021). After adjustment, the median CT-to-puncture time decreased significantly from 47 min in the pre-implementation period to 36 and 35 min in the learning and established periods, respectively (*P* < .001). Findings for CT-to-puncture remained consistently significant in PSC-restricted analyses, including when additionally stratified by working hours and off-hours.

In analyses restricted to CSCs, there was a trend towards decreased CT-to-puncture, but no statistically significant differences were observed. However, in a CSC subgroup analysis limited to working hours, CT-to-puncture decreased significantly from 29 min in the pre-implementation period to 23 and 25 min in the learning and established periods, respectively (*P* = .002/*P* = .010).

**Figure 2:**
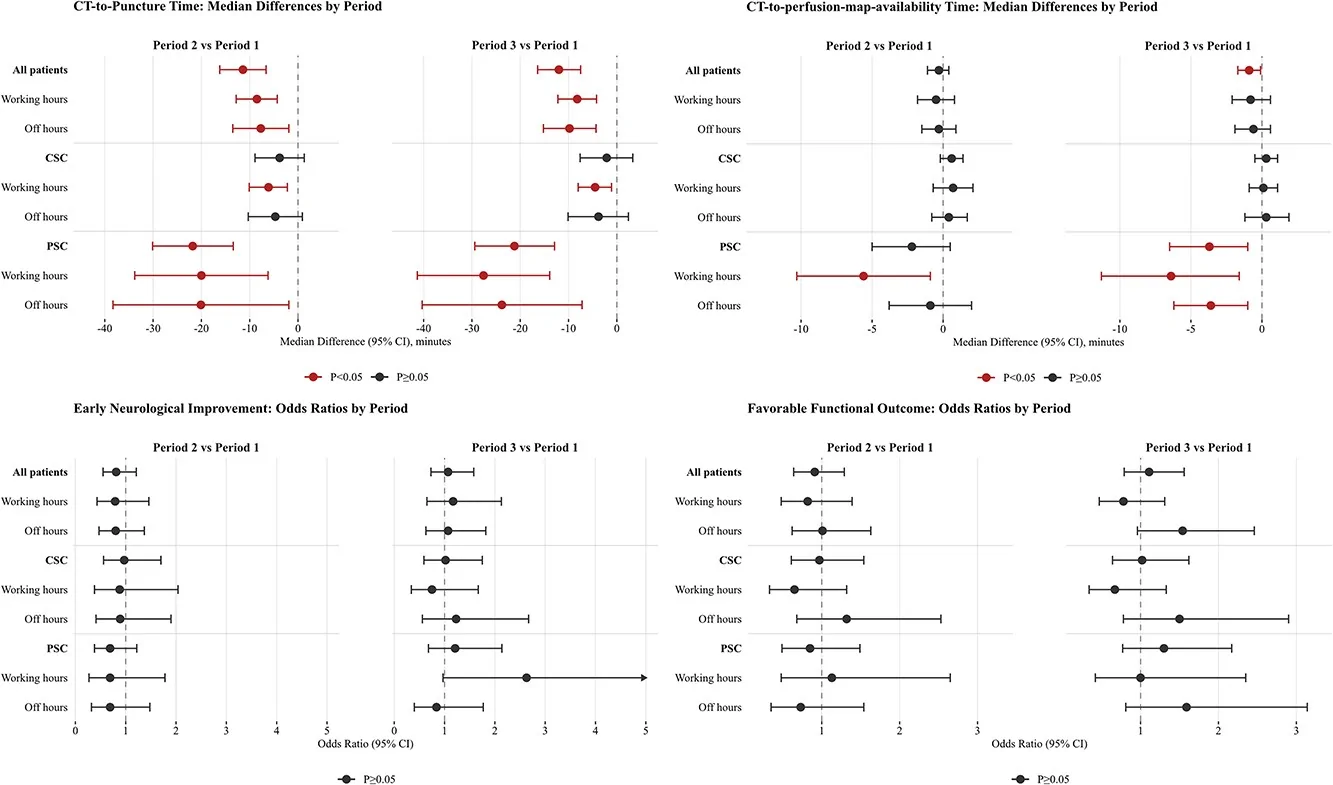
Outcomes across study periods. Negative time differences indicate shorter time intervals compared with period 1 (control period). Abbreviations: CSC = comprehensive stroke centre; OH = off-hours; PSC = primary stroke centre; WH = working-hours; Whole = whole cohort including PSC and CSC.

Adjusted organisational time metrics are presented in Figure 2 and Table S5. Unadjusted organisational time metrics are presented in Table S6.

### Clinical outcome measure

Data on early neurological outcome were available in 881 (91%) patients and of these 642 (73%) demonstrated ENI. Data on favourable functional outcome were available in 892 (92%) patients and of these 414 (46%) demonstrated FFO. No significant differences were found in clinical outcomes, adjusted results are reported in Table S7.

## Discussion

In this retrospective, registry-based study, we observed a significant reduction in both CT-to-puncture and CT-to-perfusion-map-availability times following the implementation of Brainomix 360 Stroke. Subgroup analyses indicated that these reductions were primarily driven by improvements at PSCs. Despite these organisational time gains, no corresponding differences were observed in clinical outcomes.

Our findings are consistent with previous reports demonstrating that AI-supported decision-making tools can shorten workflow times in acute stroke management. Nagaratnam et al. reported in a prospective multicentre study significantly shorter door-in-door-out time in EVT patients using Brainomix 360 Stroke (128 vs 192 min).^11^ Comparatively, our study demonstrated a significant 21-min reduction (99 vs 120 min) in CT-to-groin puncture time among PSC patients. While the observed time reductions are in line with our findings, differences in study design should be considered, as Brainomix was not applied to all patients at evaluating sites in their study. As noted by the authors, this may have introduced potential selection bias. Interestingly, they also reported higher EVT rates among patients assessed using Brainomix 360 Stroke, with the greatest benefit observed in patients presenting to regional PSCs without EVT capability. Frost et al reported a numerically shorter door-to-puncture for EVT patients (116 vs 155 min) with a significant benefit in transferred patients.^12^ Le et al. reported significantly shorter door-in-door-out time in patients presenting to a PSC in the post-implementation period.^13^ Morey et al. reported a significant reduction in door-to-neuroendovascular-team-notification time following Viz LVO (Viz.ai) implementation but did not observe a corresponding significant reduction in door-to-groin puncture time.^8^ Elijovich et al. conducted a retrospective comparison between standard imaging workflows and cases in which Viz LVO was used, demonstrating a significantly shorter CTA-to-groin puncture time among transferred patients, but not in patients presenting directly to the CSC.^14^ However, patients were retrospectively identified, and the Viz LVO group included a higher proportion of M1 occlusions and fewer tandem occlusions, suggesting potential selection bias. Matsoukas et al. used a similar design and reported a shorter PSC CTA-to-groin puncture time (146 vs 234 min) in patients where Viz LVO was used.^15^

Martinez-Gutierrez et al. reported, in a cluster randomised trial, a significant reduction of 11 min in door-to-groin time and 10 min in CT-to-EVT time in an EVT cohort of patients with direct arrival to a CSC.^3^ They evaluated Viz LVO (Viz.ai), an automated LVO detection tool based on CTA. The software automatically generated alerts to relevant clinicians and included a team communication platform for case discussion. In contrast, we did not find a significant decrease in CT-to-puncture time in patients presenting directly to the CSC. Hassan et al. reported substantial time savings of 86 min in door-to-groin puncture time among patients presenting directly to a CSC following implementation of Viz LVO.^16^ However, their pre-implementation baseline door-to-groin puncture time was 207 min, which is considerably longer than the adjusted CT-to-puncture time pre-implementation of 36 min observed at our CSC. Similarly, Figurelle et al. found a shorter door-to-groin puncture time in patients presenting directly to the CSC (86 vs 127 min).^17^ It is important to consider differences in baseline workflow efficiency when interpreting reported time reductions. Naturally, centres with longer baseline processing times before implementation have greater potential for improvement. Conversely, centres with already optimised workflows may experience more modest reductions.

In this study, we report reductions in time of internal workflow in the radiology department, possibly attributable to the integration of the AI tool into our workflow. First, AI detection of an occlusion offers confirmation of the radiologist’s assessment especially in difficult cases. Moreover, according to our regional guidelines, the radiologist on-call should not waste time reviewing the CTA extensively when no apparent occlusion is detected. Instead, the radiologist should review the CTP maps as soon as the CTP scan is completed to assess whether a perfusion defect exists indicating a missed occlusion on CTA. In the pre-implementation period, the radiologist had to manually create these maps. While this process could be completed in less than a minute, there was potential for delays due to workload or inexperience. In the post-implementation period, this process is completely automated and usually finished while the radiologist is assessing the CTA. Thus, both automated occlusion detection and AI-generated maps may have contributed to the reduction in internal workflow times observed in the post-implementation period.

In our study, the observed improvements in CT-to-puncture time were largely seen in patients initially presenting to a PSC. This finding suggests that, within an already highly optimised stroke system, the greatest gains from such interventions may be realised at lower-volume PSCs, thereby enhancing overall system efficiency. These findings underscore the need to evaluate system-wide interventions not only at high-volume CSCs but also in terms of their broader impact across the entire stroke care network.

The observed reductions in CT-to-puncture time may be attributed to multiple factors. First, the reasons described above regarding the internal radiology workflow apply here as well, as the CT-to-perfusion-map-availability time is integrated in the CT-to-puncture time. Secondly, greater access to imaging, including automated perfusion maps for the vascular neurologist and the neurointerventionist on call, may decrease post-imaging decision time by reducing diagnostic uncertainty and the need for additional communication. It has also been suggested that a faster radiological workflow and post-imaging decision-making enable ambulances to remain on site at the PSC during imaging, which may reduce the delays associated with obtaining a new ambulance for transfer. Contrary to this hypothesis, the proportion of primary ambulances remaining on site during imaging decreased in period 3 (Table S8). Several operational factors may explain this observation. When an ambulance is nearing the end of its shift or is required for another emergency before a transfer decision is reached, the crew notifies the dispatch centre, and a relief ambulance is placed on stand-by ensuring availability if transfer to the CSC is needed and thereby avoiding additional delays. This could have contributed to the fact that time-to-treatment remained significantly decreased in this period even though transfers to the CSC by the same ambulance decreased in period 3.

Despite significant time reductions no corresponding improvement in clinical outcomes was found. Several explanations are possible. First, while the time differences were statistically significant, the absolute reductions may not have been large enough to influence patient outcomes, as suggested by the a posteriori minimum detectable OR analysis, the minimum detectable OR is substantially larger than what could be expected from the magnitude of time reductions in our study.^18^ A larger study sample is likely needed to detect significant improvements in clinical outcomes. Second, stroke outcomes are multifactorial, and factors beyond workflow time—such as patient selection, collateral status, perioperative management or postoperative rehabilitation—may exert greater influence. Third, all patients were selected using perfusion imaging, and patients from regional hospitals in particular might have been selected as slow progressors who are less sensitive to pre-treatment delays. Moreover, during the study period, large, randomised trials evaluating treatment for distal occlusions and large baseline infarct cores were ongoing. We identified imbalances in these variables between the study periods and therefore adjusted for them to mitigate potential confounding, as the treatment effect in these subgroups is likely attenuated, though residual confounding might still have influenced our results. However, patients with a large baseline infarct core or distal occlusions were routinely treated at our centre prior to the study period.

Nevertheless, the possible positive influence of an AI tool introduction is not limited solely to the impact on workflow times and outcomes in treated patients. Other implications should also be considered; for example, improved identification of patients with proximal occlusions leading to an increased proportion of patients treated with EVT. Indeed, in our study, we observed a substantial increase in the number of EVTs performed in period 3 possibly attributable to the established AI tool. However, the study design did not allow for analysis of a potential association between the implementation of the AI tool and the observed increase in EVT numbers.

When considering the implementation of an AI tool for stroke imaging, the potential benefits described above must be balanced against possible risks, including increased complexity of the imaging pathway, as well as the risk of overdiagnosis and an unnecessary burden on stroke teams due to false-positive alerts. Although automated alerts may facilitate faster identification and coordination of eligible patients, excessive notifications or inaccurate alerts may contribute to alert fatigue, potentially reducing the clinical value of the system over time. Furthermore, AI-supported imaging pathways require careful integration into existing clinical workflows to ensure that they complement rather than complicate decision-making processes. Appropriate implementation strategies and continuous monitoring of performance are therefore essential to maximise benefits while minimising unintended consequences.

Multiple previous studies have evaluated the use of CTP and automated perfusion map generation for selecting patients eligible for IVT in the extended time window, contributing to the expansion of IVT eligibility. However, extended-window IVT was not part of routine clinical practice in our region during the study period, and the potential impact of the implemented software on IVT eligibility and treatment rates could therefore not be evaluated.

The strengths of our study include the use of a large, unselected clinical cohort and the inclusion of multiple PSCs in addition to our CSC (Table S9). This enhances the generalisability of our findings to other regionalised stroke care systems. Moreover, organisational changes to optimise workflow in our stroke network had already taken place in the years prior to the study period, leaving the implementation of the AI tool as the only new change implemented region-wide during the study period. Limitations include the retrospective design and lack of a control group, with their inherent susceptibility to bias and residual confounding, and the restriction to a single healthcare region, which may limit generalisability. Nevertheless, the AI tool was implemented simultaneously across the region, and taking into account that no other changes were introduced, the results presented can be expected to depict the overall implications of the AI tool.

## Conclusion

Implementation of an AI-supported decision-making tool in a high-volume stroke system was associated with significant reductions in key workflow times, largely attributable to decreased times at PSCs in the region, but was not associated with measurable improvements in clinical outcomes.

## Supplementary Material

Supplementary_data_aakag098

## Data Availability

Approval was obtained from the regional ethics committee (Dnr 2024-03780-01). Written informed consent was waived by the ethical authority. The procedures used in this study adhere to the tenets of the Declaration of Helsinki. Data are available upon reasonable request from the corresponding author, subject to ethical/privacy constraints.
