# Supplementary material for: Implementation of an AI-supported decision-making tool in a high-volume stroke system with routine perfusion imaging
Source: Eur Stroke J. 2026 Aug 20;11(8):aakag098. doi: 10.1093/esj/aakag098 (PMC13492657; doi:10.1093/esj/aakag098)
Supplement: Supplementary file 1 [file supplementary_data_aakag098.docx]

Supplementary data

**Supplementary table 1.**

| Variable | Origination | Comment |
| --- | --- | --- |
| Sex, male | SSRR (EVAS) |  |
| Age, y | SSRR (EVAS) |  |
| SBP before intervention, mmHg | SSRR (EVAS) | Baseline BP from prehospital transport. |
| Fully conscious before intervention | SSRR (EVAS) |  |
| Transfer from another hospital | SSRR (EVAS) |  |
| IV thrombolysis before intervention | SSRR (EVAS) |  |
| Stroke within 2 weeks prior | SSRR (EVAS) |  |
| Pre-stroke mRS 0-1 | SSRR (EVAS) |  |
| NIHSS before intervention | SSRR (EVAS) | Complete assessment from experienced neurologist |
| General anesthesia | SSRR (EVAS) |  |
| Referring hospital | SSRR (EVAS) |  |
| Occlusion site | SSRR (EVAS) | Location assessed by digital subtraction angiography at the start of procedure |
| Time of NCCT and CTP | Pre-implementation assessed by EC using manual extraction of DICOM data, post-implementation by brainomix server data |  |
| Time of puncture | SSRR (EVAS) |  |
| Neurological outcome at 24 hours | SSRR (EVAS) |  |
| Functional outcome at 3 months | SSRR (from RIKSSTROKE registry using questionnaire or telephone interview). In case of missing, assessed using electronic medical records by experienced vascular neurologist |  |

**Validity**

**EVAS (Adapted from registry web site^1^):**

EVAS is governed by a Steering Group with members representing every thrombectomy center in Sweden (neurologist and neurointerventionist from every center) including representation from Riksstroke registry and from the Swedish patient stroke association. EVAS includes also coordinating nurses, statisticians and a data manager.

Validation of registry data: The EVAS register performs continuous validation of data, both automatically and manually.

*Automatic validation*

During data entry, automatic checks are performed against predefined maximum and minimum values. The data entry forms are conditional, meaning that only information relevant to the specific treatment modality is collected. This has minimized the number of missing values.

*Manual validation*

Manual validation of data is carried out quarterly. This validation includes logical consistency checks and cross-validation of related variables. Date and time entries are reviewed for plausibility. Loss to follow-up is assessed and reported in the annual report.

*Follow-up of deviations*

Each participating center receives information on deviations identified during the quarterly validation as well as during the compilation of the annual report.

**Riksstroke (Adapted from registry website^2^):**

Riksstroke is governed by a Steering Group with members representing expertise in stroke care (neurology, internal medicine, geriatrics, nursing, physiotherapy), epidemiology and administration. The Swedish patient stroke association is also represented in the Steering Group. Riksstroke is the national quality register for stroke care in Sweden and one of the largest of the many quality assessment registers in the country. It was initiated in 1994 with the primary aim to monitor and improve the quality of stroke care and research. Since 1998 the register includes all hospitals admitting patients with acute stroke in Sweden.

The Riksstroke Coordinating Centre includes coordinating nurses, statisticians and a data manager. The centre collects data from and provides support and structured feedback to the hospitals participating in Riksstroke, performs quality controls and validations, coordinates the work performed at a supporting computer centre, performs analyses and produces annual reports. It also supports scientific work based on Riksstroke data.

*Data capture and handling*

All acute stroke incidents in hospitals are to be registered. Patients diagnosed with ischemic stroke (ICD-10 I63), intracerebral haemorrhage (ICD-10 I61) or unspecified acute cerebrovascular event (ICD-10 I64) are eligible for registration in Riksstroke.

Data is usually collected through completion of printed forms during the hospital stay. At 3 months after stroke, patients are asked to respond to a questionnaire by letter, phone or at a return visit. If the patient is unable to respond, the questionnaire is filled in by a next-of-kin or by healthcare/social service staff.

The recorded data is transferred to the Riksstroke data base managed by Umeå University computer center. Control functions to ensure the accuracy of personal identity number, sex, dates, etc., are embedded in the data entry software and only valid response alternatives are accepted.

*Validity*

Data quality is ensured through both automated validation checks performed during data entry via the web-based registration form and statistical process control, whereby alerts for outlying results are subsequently reviewed manually to determine whether they reflect actual errors or natural variation. Following each manual review of outlying values, a validation report is prepared documenting the deviation, the validation process undertaken, and any corrective actions implemented.

Hospital-specific coverage rates and the automated validation checks applied during data entry are reviewed regularly and updated or revised as needed (e.g., following modifications to the registration forms). Additional validation studies of the Swedish Stroke Register (Riksstroke), including comparisons with medical records, have been published previously (Buchwald F, Ström JO, Norrving B, Petersson J. Validation of Diagnoses of Transient Ischemic Attack in the Swedish Stroke Register (Riksstroke) TIA Module. Neuroepidemiology. 2015;45:40–43; Söderholm A, Stegmayr B, Glader EL, Asplund K; Riksstroke Collaboration. Validation of Hospital Performance Measures of Acute Stroke Care Quality in Riksstroke, the Swedish Stroke Register. Neuroepidemiology. 2016;46:229–234).

**Supplementary table 2.**

| Improvements in the stroke workflow before 2022 | |
| --- | --- |
| *General* | Acute stroke patients are transported directly to CT-lab, bypassing emergency department. |
|  | Assembly of neurology and radiology teams in CT room before patient arrival |
|  | Annual simulation training from arrival at CT room to groin puncture |
| *Ambulance* | Pre-hospital standardized triage method and pre-notification of local stroke team |
|  | Expanding CSC catchment area for direct transport (45-minute transport radius) |
|  | Standardized training program for pre-hospital personal (modified NIHSS, communication) |
|  | Introduction of "thrombectomy alarm" in CSC (direct and referral cases) |
|  | Ambulance stays on standby at referral hospital for 20 min in case of secondary transport to CSC |
|  | Initiation of secondary transport prior to consultation with CSC |
| *Neurology* | Standardized NIHSS evaluation in CT room |
|  | Neurology team accompanies patient from CT to angiosuite |
|  | Thrombolysis nurse in the CT room in all incoming cases |
|  | Mandatory thrombolysis certification for all neurology staff involved in stroke |
|  | Establishment of CSC regional stroke neurologist 24/7 |
| *Radiology* | Standby CT scanner upon stroke alarm |
|  | CT interpretation and report of findings is performed in parallel with neurological assessment in the CT room |
|  | Unified stroke diagnostic protocols in all regional hospitals |
|  | Addition of CT Angiography and CTP for all acute stroke cases in all stroke centers in the region |
| *Anesthesiology* | Standardized sedation and general anesthesia protocol. Pre-filled medication trays prepared in advance |
|  | Invasive arterial blood pressure monitoring |
|  | Fixed work positions of neurointerventional, anesthesia and neurology teams to facilitate parallel work |
|  | Multidisciplinary decision on general anesthesia or conscious sedation in angiosuite |
| *Neurointerventional* | Dedicated thrombectomy telephone line |
|  | Introduction of starting stroke kit for groin puncture and initial angiography |
|  | Standardized EVT material |
|  | Consensus on EVT procedure method among neurointerventionists |
|  | Ultrasound-guided punctures |

**Supplementary table 2**: Improvement in the stroke workflow before 2022. All changes were introduced from 2010 to 2018. From 2018 to current year an annual simulation is taking place, but no organizational changes have been introduced since then.

Description of the workflow:

Prehospital: Ambulance dispatched at the patient’s location. If a stroke is suspected the nearest PSC stroke physician in charge is contacted or the regional stroke neurologist at CSC if the location of the patient is within 45 min transport radius from the CSC. The physician in charge triggers the stroke alarm when the ambulance is within 15 minutes transport time from the PSC or CSC. The radiology staff places a CT scanner on standby, and both the radiologist and thrombolysis team assemble at the CT scanner where the physician in charge reports the case.

Intrahospital: The ambulance bypasses the emergency department and transports the patient to the CT scanner. The physician in charge starts the neurological evaluation and the radiology staff prepares for the NCCT scan. The neurological evaluation is interrupted if the radiology staff is ready for the first scan and continuous immediately afterwards while the radiologist review the NCCT and the radiology staff prepare for CTA and CTP. Intravenous thrombolysis is given as soon as the radiologist excludes radiological contraindications and no other medical contraindications exist, before or during CTA and CTP acquisition. The radiologist on-call should not waste time reviewing the CTA extensively when no apparent occlusion is detected. Instead, the radiologist should review the CTP maps as soon as the CTP scan is completed to assess whether a perfusion defect exists indicating a missed occlusion in CTA. Since June 2022, when Brainomix 360 Stroke is introduced, the radiologist, have already logged in in the application and review the AI-tool´s report for every scan in parallel with his/her assessment. If a large vessel occlusion is detected (by the radiologist or the AI tool) the physician in charge will contact the CSC (or the neurointerventionist if the case is already at the CSC) if the patient presents within 6 hours from stroke onset. If the patient presents in the 6-24 h window or has a large infarct core, the CSC (or the neurointerventionist respectively) is contacted when also the CTP is performed and show favorable profile. The ambulance crew transporting the patient to the PSC are waiting 20 minutes after arrival at the CT scanner for possible secondary transfer to the CSC. In case that the same cannot wait (ending of shift or dispatched to another nearby emergency case) the dispatch center is notified to place a relief ambulance on standby in case it is needed for transfer to CSC.

CSC (neurointervention): If the patient is transferred from a PSC, the ambulance crew contacts the CSC stroke neurologist in charge 15 minutes before arrival and the stroke neurologist triggers the thrombectomy alarm. The anesthesiology and neurointerventional team assemble in the neuroangiography suite and the stroke neurologist reports the case. Decision on anesthesia form is made at this point. If the patient is already in the CSC (as direct transport to the CSC radiology department) then the stroke neurologist triggers the thrombectomy alarm as soon as a decision is reached I the CT scanner of the CSC. The anesthesiology and neurointerventional team assemble in the neuroangiography suite starting preparations. When the neurology team arrives with the patient, the anesthesia form is decided after a brief report and discussion between the neurologist, the anesthesiologist and neurointerventionist while the rest of the teams are preparing the patient.

Procedure: Neurointerventional and anesthesiology team are working in parallel; then neurointerventional team is working on the patient´s right side and prepare for groin puncture while the anesthesiology team works on patient´s left side and head.

Detailed workflow can be found in the films provided in the following location “<https://larportalen.vgregion.se/course/view.php?id=3831>” or <https://larportalen.vgregion.se/> and choose log in as guest and search under “utbudskatalog” = ”VGR strokeutbildning”

**Supplementary Table 3.**

| Group | Outcome | N | Event rate | Min OR |  |
| --- | --- | --- | --- | --- | --- |
| Whole | ENI | 889 | 0.717 | 1.56 |  |
| Whole | FFO | 905 | 0.454 | 1.46 | |
| CSC | ENI | 501 | 0.743 | 1.88 |  |
| CSC | FFO | 512 | 0.477 | 1.65 |  |
| PSC | ENI | 388 | 0.683 | 1.94 |  |
| PSC | FFO | 393 | 0.425 | 1.77 |  |

**Supplemental Table 3.** A posterior minimum detectable OR analysis. CSC, Comprehensive Stroke Center; ENI, Early Neurological Improvement; FFO, Favorable Functional Improvement; PSC, Primary Stroke Center.

**Supplementary Table 4.**

| Outcome | Cohort | Period comparison | Multiple Imputation (min) | Complete Case (min) |
| --- | --- | --- | --- | --- |
| CT-to-CTP | Whole | Period 2 vs Period 1 | -0.34 | -0.22 |
|  | Whole | Period 3 vs Period 1 | -0.89 | -0.84 |
|  | CSC | Period 2 vs Period 1 | 0.56 | 0.60 |
|  | CSC | Period 3 vs Period 1 | 0.28 | 0.24 |
|  | PSC | Period 2 vs Period 1 | -2.22 | -2.48 |
|  | PSC | Period 3 vs Period 1 | -3.73 | -3.97 |
| CT-to-puncture | Whole | Period 2 vs Period 1 | -11.39 | -11.54 |
|  | Whole | Period 3 vs Period 1 | -11.97 | -12.44 |
|  | CSC | Period 2 vs Period 1 | -3.80 | -5.00 |
|  | CSC | Period 3 vs Period 1 | -2.13 | -2.37 |
|  | PSC | Period 2 vs Period 1 | -21.76 | -22.00 |
|  | PSC | Period 3 vs Period 1 | -21.17 | -21.79 |

**Supplementary Table 4.** Multiple imputation vs complete case comparison. CSC, Comprehensive Stroke Center; PSC, Primary Stroke Center.

**Supplementary Table 5.**

| **CT-to-Puncture Time** |  |  |  |  |  |  |
| --- | --- | --- | --- | --- | --- | --- |
| **Group** | **Period 1 Adjusted Median (95% CI), min** | **Period 2 Median Difference (95% CI), min** | **P Value** | **Period 3 Median Difference (95% CI), min** | **P Value** | **N** |
| **All patients** | **47.4 (39.6, 55.3)** | **-11.4 (-16.2, -6.6)** | **0.000** | **-12 (-16.4, -7.5)** | **0.000** | **921** |
| Working hours | 33.6 (29.6, 37.6) | -8.5 (-12.8, -4.3) | 0.000 | -8.2 (-12.2, -4.2) | 0.000 | 415 |
| Off hours | 68 (55.5, 80.4) | -7.7 (-13.5, -1.9) | 0.009 | -9.8 (-15.2, -4.3) | 0.000 | 506 |
| **CSC** | **36.2 (31.8, 40.6)** | **-3.8 (-8.9, 1.3)** | **0.148** | **-2.1 (-7.6, 3.3)** | **0.445** | **510** |
| Working hours | 29 (26.2, 31.8) | -6.1 (-10.1, -2.2) | 0.002 | -4.5 (-8, -1.1) | 0.010 | 254 |
| Off hours | 46.4 (41.7, 51.2) | -4.7 (-10.3, 0.9) | 0.098 | -3.8 (-10.1, 2.4) | 0.229 | 256 |
| **PSC** | **119.8 (111.5, 128.1)** | **-21.8 (-30.1, -13.4)** | **0.000** | **-21.2 (-29.4, -12.9)** | **0.000** | **411** |
| Working hours | 120 (108.5, 131.4) | -20 (-33.8, -6.2) | 0.005 | -27.6 (-41.3, -13.9) | 0.000 | 161 |
| Off hours | 133.5 (115, 152) | -20.1 (-38.3, -1.9) | 0.031 | -23.8 (-40.3, -7.2) | 0.005 | 250 |

| **CT-to-CTP Time** |  |  |  |  |  |  |
| --- | --- | --- | --- | --- | --- | --- |
| **Group** | **Period 1 Adjusted Median (95% CI), min** | **Period 2 Median Difference (95% CI), min** | **P Value** | **Period 3  Median Difference (95% CI), min** | **P Value** | **N** |
| **All patients** | **7 (6.2, 7.7)** | **-0.3 (-1.1, 0.4)** | **0.393** | **-0.9 (-1.7, -0.1)** | **0.025** | **879** |
| Working hours | 6.6 (5.1, 8) | -0.5 (-1.8, 0.8) | 0.474 | -0.8 (-2.1, 0.6) | 0.265 | 395 |
| Off hours | 7 (6.1, 7.9) | -0.3 (-1.5, 0.9) | 0.629 | -0.6 (-1.9, 0.6) | 0.302 | 484 |
| **CSC** | **5.2 (4.5, 6)** | **0.6 (-0.2, 1.4)** | **0.172** | **0.3 (-0.5, 1.1)** | **0.508** | **495** |
| Working hours | 5.2 (4.3, 6) | 0.7 (-0.7, 2.1) | 0.309 | 0.1 (-0.9, 1.1) | 0.853 | 242 |
| Off hours | 6 (5.1, 6.8) | 0.4 (-0.8, 1.7) | 0.505 | 0.3 (-1.2, 1.9) | 0.653 | 253 |
| **PSC** | **9.4 (6.7, 12.2)** | **-2.2 (-5, 0.5)** | **0.115** | **-3.7 (-6.5, -1)** | **0.008** | **384** |
| Working hours | 13.1 (8.7, 17.6) | -5.6 (-10.3, -0.9) | 0.019 | -6.4 (-11.3, -1.6) | 0.009 | 153 |
| Off hours | 9.4 (6.7, 12.1) | -0.9 (-3.8, 2) | 0.545 | -3.6 (-6.2, -1) | 0.006 | 231 |

**Supplementary table 5 – Adjusted organizational outcomes.** Period 1 (Control period) is presented as adjusted median (95% confidence interval) and serves as the reference. Periods 2 and 3 are expressed as adjusted median differences versus Period 1. Negative time differences indicate shorter time intervals. CTP, CT perfusion; CSC, comprehensive stroke center; PSC, primary stroke center.

**Supplementary Table 6**

| Unadjusted medians by period, group, and time of day | | | | | |
| --- | --- | --- | --- | --- | --- |
| **Outcome** | **Group** | **Time of day** | **Period 1 Median (95% CI)** | **Period 2 Median (95% CI)** | **Period 3 Median (95% CI)** |
| CT-to-punction (min) | Whole | All | 70 (56, 79) | 49.2 (44.2, 60.2) | 60.1 (52.4, 67.1) |
|  |  | Working hours | 38 (33, 62) | 30.8 (27.2, 39.9) | 37 (28.8, 60.1) |
|  |  | Off hours | 80 (67, 94) | 62.9 (52.8, 75) | 69.5 (59.3, 85.9) |
|  | CSC | All | 38 (34, 42) | 33.5 (29.8, 36.3) | 35.3 (31.6, 37) |
|  |  | Working hours | 29 (27, 31) | 24.3 (23, 26.8) | 23.7 (21.9, 26.4) |
|  |  | Off hours | 48 (44, 50) | 44.2 (41, 46.5) | 45.3 (40, 48.3) |
|  | PSC | All | 132 (119, 150) | 102.9 (94.9, 115.1) | 96 (92.2, 102) |
|  |  | Working hours | 122 (101, 132) | 100.5 (87.9, 115.1) | 92.9 (84.3, 97.5) |
|  |  | Off hours | 149.5 (117, 165) | 110.1 (94.9, 133.4) | 102 (92.9, 110.2) |
| CT-to-CTP (min) | Whole | All | 7 (6, 8) | 6.9 (6, 7.2) | 6 (5.4, 6.5) |
|  |  | Working hours | 7 (6, 8.5) | 6.4 (5.9, 7.3) | 5.9 (5.2, 6.4) |
|  |  | Off hours | 7 (6, 8) | 7 (6, 7.9) | 6.4 (5.3, 7) |
|  | CSC | All | 6 (5, 6) | 6 (5.6, 7) | 5.9 (5.3, 6.6) |
|  |  | Working hours | 5 (5, 6) | 6.3 (5.5, 7.2) | 5.4 (5, 6.4) |
|  |  | Off hours | 6 (5, 6) | 5.8 (5, 7.3) | 6.5 (5.7, 7.3) |
|  | PSC | All | 11 (9, 14) | 7.2 (6.8, 8.7) | 6.1 (5.2, 6.9) |
|  |  | Working hours | 14 (9, 17) | 6.7 (5.8, 8.6) | 6.1 (5.4, 7.3) |
|  |  | Off hours | 10 (8, 13) | 8 (7, 10.9) | 6.3 (5, 7.2) |

**Supplementary Table 6.** Unadjusted median time by period, group and time of day. CSC, Comprehensive Stroke Center; PSC, Primary Stroke Center.

**Supplementary table 7.**

| **Early Neurological Improvement** | | | | | |
| --- | --- | --- | --- | --- | --- |
| **Group** | **Period 2 OR (95% CI)** | **P Value** | **Period 3 OR (95% CI)** | **P Value** | **N** |
| **All patients** | **0.80 (0.54, 1.19)** | **0.265** | **1.06 (0.72, 1.56)** | **0.766** | **881** |
| Working hours | 0.77 (0.42, 1.42) | 0.408 | 1.18 (0.65, 2.13) | 0.589 | 393 |
| Off hours | 0.79 (0.47, 1.34) | 0.386 | 1.04 (0.62, 1.76) | 0.879 | 488 |
| **CSC** | **0.94 (0.54, 1.63)** | **0.826** | **1.02 (0.59, 1.75)** | **0.949** | **497** |
| Working hours | 0.84 (0.37, 1.91) | 0.672 | 0.76 (0.34, 1.67) | 0.489 | 242 |
| Off hours | 0.91 (0.43, 1.94) | 0.811 | 1.21 (0.56, 2.60) | 0.631 | 255 |
| **PSC** | **0.68 (0.38, 1.20)** | **0.184** | **1.20 (0.68, 2.12)** | **0.529** | **384** |
| Working hours | 0.72 (0.28, 1.85) | 0.492 | 2.65 (0.98, 7.19) | 0.055 | 151 |
| Off hours | 0.69 (0.32, 1.47) | 0.336 | 0.84 (0.40, 1.75) | 0.643 | 233 |

| **Favorable Functional Outcome** | | | | | |
| --- | --- | --- | --- | --- | --- |
| **Group** | **Period 2 OR (95% CI)** | **P Value** | **Period 3 OR (95% CI)** | **P Value** | **N** |
| **All patients** | **0.89 (0.63, 1.26)** | **0.516** | **1.09 (0.77, 1.53)** | **0.631** | **892** |
| Working hours | 0.78 (0.47, 1.32) | 0.360 | 0.80 (0.48, 1.34) | 0.397 | 402 |
| Off hours | 1.01 (0.62, 1.63) | 0.968 | 1.47 (0.92, 2.34) | 0.104 | 490 |
| **CSC** | **0.95 (0.60, 1.51)** | **0.829** | **1.02 (0.64, 1.61)** | **0.945** | **501** |
| Working hours | 0.60 (0.30, 1.19) | 0.145 | 0.68 (0.34, 1.34) | 0.266 | 248 |
| Off hours | 1.37 (0.72, 2.63) | 0.340 | 1.48 (0.77, 2.85) | 0.237 | 253 |
| **PSC** | **0.83 (0.48, 1.43)** | **0.499** | **1.22 (0.73, 2.04)** | **0.438** | **391** |
| Working hours | 1.14 (0.49, 2.66) | 0.757 | 1.00 (0.43, 2.33) | 0.996 | 154 |
| Off hours | 0.67 (0.32, 1.41) | 0.297 | 1.44 (0.74, 2.79) | 0.285 | 237 |

**Supplementary table 7 – Adjusted clinical outcomes.** Periods 2 and 3 are expressed as adjusted Odds Ratios (OR) versus Period 1 (Control period). Negative OR indicate worse clinical outcome. CTP, CT perfusion; CSC, comprehensive stroke center; PSC, primary stroke center.

**Supplementary table 8.**

| Period | Total (n) | Secondary transfer with same ambulance (n) | Percentage (%) |
| --- | --- | --- | --- |
| Period 1 | 128 | 80 | 63 |
| Period 2 | 111 | 71 | 64 |
| Period 3 | 170 | 89 | 52 |

**Supplementary table 3.** Secondary transfer with same ambulance as initial transport to primary stroke center, per period (PSC). Total number equals all patients with initial transfer to PSC.

**Supplementary table 9.**

| CSC | Sahlgrenska University Hospital |
| --- | --- |
| PSC 1 | Skaraborgs Sjukhus i Skövde |
| PSC 2 | Skaraborgs Sjukhus i Lidköping |
| PSC 3 | Norra Älvsborgs Länssjukhus |
| PSC 4 | Södra Älvsborgs Sjukhus |
| PSC 5 | Alingsås Lasarett |
| PSC 6 | Kungälvs Sjukhus |

**Supplementary table 9.** Abbreviations and corresponding stroke centers

1.	EVAS. Registret för endovaskulär behandling av stroke. <https://evas-registret.se/sample-page/forskning/datavalidering/>. Accessed 28 June.

2.	Riksstroke. <https://www.riksstroke.org/sve/omriksstroke/allman-information/>. Accessed 28 June.
